# Draft-genome sequences of *Leptospira santarosai* strains isolated from urogenital tract of cows

**DOI:** 10.1128/MRA.00686-23

**Published:** 2023-11-03

**Authors:** Maria Isabel Nogueira Di Azevedo, Frederico Kremer, Camila Ezepha, Gabriela Bergiante Kraychete, Pascale Bourhy, Walter Lilenbaum

**Affiliations:** 1 Fluminense Federal University, Laboratory of Veterinary Bacteriology, Biomedical Institute, Niterói, Rio de Janeiro, Brazil; 2 Technological Development Center, Federal University of Pelotas, Capão do Leão, Rio Grande do Sul, Brazil; 3 Laboratory of Investigation in Medical Microbiology (LIMM), Paulo de Góes Institute of Microbiology, Federal University of Rio de Janeiro, Rio de Janeiro, Brazil; 4 National Reference Center for Leptospirosis, Biology of Spirochetes Unit, Institut Pasteur, Paris, France; Indiana University, Bloomington, Bloomington, Indiana, USA

**Keywords:** leptospirosis, vaginal fluid, cattle, bovine

## Abstract

*Leptospira santarosai* serovar Guaricura is adapted to bovines and is associated with a chronic disease that causes reproductive disorders, leading to important economic losses. Here, we present the draft genomes of three *L. santarosai* strains isolated from vaginal fluid and one from the urine of cows with reproductive disorders from Brazil.

## ANNOUNCEMENT

Leptospirosis is a neglected zoonosis caused by bacteria of the genus *Leptospira* and affects domestic and wild animals and humans (1). According to genetic classification, leptospiral species are currently divided into approximately 68 genomospecies separated into pathogenic (subclade P1), intermediate (subclade P2), and saprophytic (subclades S1 and S2) (2). In the pathogenic group, *Leptospira santarosai* from Sejroe serogroup, serovar Guaricura, is recognized as an important agent of bovine genital leptospirosis (BGL), a chronic and silent disease that causes reproductive disorders such as abortions, stillbirths, estrus repetition, and economic losses (3).

Despite clinical signs related to reproductive failure, most studies have focused on detecting viable leptospires or their DNA in urine (3). As such, little is known about the pathophysiology of leptospires in the reproductive tract, and there is a lack of information about important aspects of BGL. Based on this, we performed whole-genome sequencing (WGS) of three strains from *L. santarosai* Sejroe serogroup—2013_VF52, 2014_VF66, and VF237—isolated from vaginal fluid of cows with reproductive injuries from Rio de Janeiro, Brazil, previously characterized by Loureiro et al. (4) (2013_VF52 and 2014_VF66) and by Aymée et al. (5) (VF237). Additionally, the strain 2014_U76 (*L. santarosai* Sejroe serogroup), previously isolated from the urinary tract of a cow (4), was also submitted to WGS. Briefly, after collection, samples were seeded into tubes with EMJH medium supplemented with the antimicrobial cocktail STAFF (6). Tubes were maintained at room temperature and transported to the laboratory. All cultures were maintained at 29°C and examined weekly with dark field microscopy for 16 weeks. Sequences were then deposited in the Collection of Bacterial Cultures of Veterinary Interest (Federal Fluminense University, Brazil).

High-quality genomic DNA was obtained from 5 × 10^10^ to 2 × 10^11^ pelleted *L. santarosai* cultures. DNA was extracted using the Dneasy Blood and Tissue Kit (Qiagen, California, USA), according to the manufacturer’s instructions. WGS was performed on Illumina (San Diego, California, USA) MiSeq platform with a 150 × 2-bp run starting from a genomic library prepared using a Nextera DNA Flex Kit (Illumina).

Reads were quality evaluated using fastQC, and all four genomes were *de novo* assembled using the Unicycler 0.4.8 (7) workflow, applying default parameters. The annotation was added by the NCBI Prokaryotic Genome Annotation Pipeline. Descriptive information regarding the sequenced genomes is summarized in Table 1.

**TABLE 1:** Four *Leptospira santarosai* Sejroe serogroup genomes released to NCBI

Isolate ID	Accession no.	Source	Read length (bp)	No. of reads^*a*^	Sequencing depth (%)	Genome size (bp)	No. of contigs (≥100 bp)	N50 (bp)	GC content (%)	No. of CDSs^*b*^	No. of tRNAs	No. of rRNAs	No. of ncRNAs	CRISPR arrays
2013_VF52	JAUOTG000000000	Cow	143	2.8	100.6	3,980,734	115	100,276	41.84	3,571	37	6	2	4
2014_VF66	JAUOTF000000000	Cow	144	2.6	94.4	3,964,329	105	114,588	42.00	3,596	37	4	2	4
VF237	JAUOTH000000000	Cow	147	4.8	177.2	3,980,991	113	91,177	41.85	3,565	37	6	2	4
2014_U76	JAUOTE000000000	Cow	144	2.4	84.9	4,069,816	140	97,505	41.83	3,711	37	6	2	5

^*a*^ These values include both paired-end reads.

^*b*^ CDSs, coding DNA sequences.

## Data Availability

All four genomes have been deposited in GenBank under the accession numbers listed in Table 1. Additionally, data are available at NCBI BioProject (PRJNA996652) and NCBI BioSample (SAMN36620640, SAMN36620641, SAMN36620642, and SAMN36620643). Raw sequencing data in FASTQ format were submitted to NCBI SRA and are available under the accession codes SRR26260710, SRR26260711, SRR26260712, and SRR26260713.
